# Sonochemical synthesis of a copper reduced graphene oxide nanocomposite using honey and evaluation of its antibacterial and cytotoxic activities

**DOI:** 10.3389/fmolb.2022.995853

**Published:** 2022-09-30

**Authors:** Nur Afini Ismail, Kamyar Shameli, Siti Nur Amalina Mohamad Sukri, Hirofumi Hara, Sin-Yeang Teow, Hassan Moeini

**Affiliations:** ^1^ Malaysia-Japan International Institute of Technology, Universiti Teknologi Malaysia Jalan Sultan Yahya Petra, Kuala Lumpur, Malaysia; ^2^ Department of Biotechnology, Graduate School of Agricultural and Life Sciences, University of Tokyo, Tokyo, Japan; ^3^ School of Medical and Life Sciences (SMLS), Sunway University, Kuala Lumpur, Malaysia; ^4^ School of Medicine, Institute of Virology, Technical University of Munich, Munich, Germany

**Keywords:** sonochemical method, copper/reduced graphene oxide nanocomposite, honey, antibacterial, cytotoxicity assay

## Abstract

The combination of graphene-based materials and inorganic nanoparticles for the enhancement of the nanomaterial properties is extensively explored nowadays. In the present work, we used a sonochemical method to synthesize a copper/reduced graphene oxide (Cu/RGO) nanocomposite using Australian honey and vitamin C as capping and reducing agents, respectively. The honey-mediated copper/reduced graphene oxide (H/Cu/RGO) nanocomposite was then characterized through UV-visible, XRD, HRTEM, and FTIR analysis. The copper nanoparticles (Cu-NPs) in the nanocomposite formed uniform spherical shapes with a size of 2.20 ± 0.70 nm, which attached to the reduced graphene oxide (RGO) layers. The nanocomposite could suppress bacterial growth in both types of bacteria strains. However, in this study, the nanocomposite exhibited good bactericidal activity toward the Gram-positive bacteria than the Gram-negative bacteria. It also showed a cytotoxic effect on the cancer colorectal cell line HCT11, even in low concentrations. These results suggested that the H/Cu/RGO nanocomposite can be a suitable component for biomedical applications.

## Introduction

In the past 50 years, pathogenic bacteria have caused a plethora of diseases in the human population. Some of these major emerging bacteria include *Staphylococcus aureus* (*S. aureus*), *Escherichia coli* (*E. coli*), *Clostridium difficile*, *Campylobacter spp*., and *Helicobacter pylori* (Vouga and Greub, 2016). Although some commensal bacteria such as *Lactobacillus* and *Bifidobacterium* may exist as part of the human microbiota, which might play beneficial roles in maintaining homeostasis (Wang et al., 2017), many of these bacteria could also cause various illnesses such as liver diseases, infection, respiratory diseases, gastrointestinal malignancy, and metabolic disorders (Wang et al., 2017). The emergence of bacterial antimicrobial resistance (AMR) has even aggravated this issue and poses a major threat to global health. According to a recent systematic analysis, the six leading pathogens responsible for death associated with AMR are *E. coli*, *S. aureus*, *Klebsiella pneumoniae*, *Pseudomonas aeruginosa (P. aeruginosa)*, *Streptococcus pneumoniae*, and *Acinetobacter baumannii* which are the combination of both Gram-positive and Gram-negative strains (Murray et al., 2022).

The second main cause of death in the United States is cancer, and among different cancer types, lung cancer is the leading cause of cancer mortality (Siegel et al., 2022). For both sexes, the highest incidence of cancer is led by breast cancer, followed by prostate and lung/bronchus cancers (Siegel et al., 2022). Similarly, chemo- and/or radio-resistance presented by the patient’s tumor remains the main barrier to effectively eradicating tumor from the body. On top of this, off-target side effects suffered by the patients due to cancer therapy are another obstacle (Miller et al., 2022). Hence, it is vital to look for a novel anticancer drug or therapeutic strategy to treat cancer more effectively.

Recently, nanomaterial has been widely explored for their special properties to mitigate these problems. There are various factors that may affect the biological activities of the nanomaterial, such as the shape, size, electronic structure, surface properties, and some additional factors related to the interaction conditions between the materials and the target cells (Sengupta et al., 2019). Copper nanoparticles (Cu-NPs) have been widely assessed for their properties. Aside from its low-cost production, copper also exhibits good thermal and electrical conductivity, and biological and antimicrobial activities (Zhou et al., 2019; Noman et al., 2020; Merugu et al., 2021). Recently, the United States Environmental Protection Agency recognized copper as the first solid antimicrobial material (Ouyang et al., 2013; Arendsen et al., 2019). However, researchers found that pure metallic Cu-NPs are difficult to obtain as copper tends to oxidize easily when exposed to the air, and it will also tend to agglomerate without proper protection (Rostami-Tapeh-Esmaeil et al., 2021). Hence, the usage of green material as a capping agent or stabilizer has gained researchers’ attention since it is reported to produce monodispersed pure Cu-NPs by a fast and green method (Nagar and Devra, 2018).

Graphene is a unique structure that attracts great attention due to its interesting physical and chemical characteristics (Luo et al., 2020), including large surface area, good conductivity, and high thermal properties (Ouyang et al., 2013). Graphene and its derivatives are used in extensive applications such as electronic devices (Moozarm Nia et al., 2017), energy storage (Rawal et al., 2020), and biomedical applications (Kumar et al., 2017). The presence of oxygen functional groups such as epoxide, carboxyl, and hydroxyl in the structure of the graphene oxide (GO) and reduced graphene oxide (RGO) makes them suitable for the production of nanocomposites (Gan et al., 2019a). These groups act as bioactive molecules that could functionalize the graphene sheet with other materials such as metal and metal oxide nanoparticles (Gan et al., 2019b; Jang et al., 2020).

Graphene oxide can be decorated with some materials through chemical reduction (*in situ*), hydrothermal, and electrochemical processes, and through the attachment of the premade nanoparticles to the graphene surface (*ex situ*) to form graphene-based nanomaterials (Sarkar and Dolui, 2015; Yin et al., 2015; Iranshahi and Iranshahi, 2022; Thy et al., 2022). Luo et al. (2020) used refluxed process in synthesizing the reduced graphene oxide/copper nanocomposites (RGO/Cu-NCs) in an oil bath at 100°C for 24 h with hydrazine hydrate. This method involved a long processing time and hazardous material. In fact, nowadays, researchers are interested in using simple and green materials to synthesize the Cu/RGO nanocomposite since it is a much eco-friendlier method. Rios et al. (2019) used an *in situ* reduction method to produce reduced graphene oxide/copper nanoparticles (RGO/Cu-NPs) in the presence of ascorbic acid for 12 h at 80°C. Fahiminia et al. (2019) synthesized Cu/RGO nanocomposites using plant extract (*Euphorbia cheiradenia Boiss*) and applied it as a catalyst for dye removal. Yang et al. (2019) produced cuprous oxide/reduced graphene oxide (Cu_2_O-RGO) nanocomposites through chemical reduction by using polyethylene glycol (PEG) and ascorbic acid with the addition of sodium hydroxide (NaOH), and used them for the antibacterial study.

Indeed, Tu et al. (2021) reported that the Cu/RGO nanocomposite exhibited better biological activity compared to the reduced graphene oxide (RGO) alone. Generally, the combination of RGO and copper ions happened by the cation- π interaction between copper ions and π -electrons that coming from the aromatic rings of RGO (Xu et al., 2019; Yan et al., 2019; Ismail et al., 2021; Tu et al., 2021). This functionalized RGO could enhance the antibacterial activity where both participated in killing the bacteria cells through electrostatic interaction between the positive charge of copper ions from the nanocomposite and the negatively charged membranes of bacteria (Sanchez-Lopez et al., 2020). RGO could also kill bacteria through the sharp edge of its structure (Prasad et al., 2017). The aggregation of the RGO due to the π – π stacking would have a hydrophobic structure which is known to give strong bacteria absorption that could help in better releasing copper ions and attacking the bacteria cell efficiently (Szunerits and Boukherroub, 2016). This will show excellent results in antibacterial activity compared to the copper ion and RGO alone. Up until now, few studies for anticancer using Cu/RGO nanocomposite were reported. Kodous et al. (2022) found that Cu/RGO nanocomposites produced by using the ultrasonication method could inhibit human breast cancer cells (MCF-7 cancer cells).

Honey is considered a green material since it is a non-toxic substance that possesses rich sugar source carbohydrate components (Balasooriya et al., 2017). It is also a simple material that does not have to undergo any extraction process, unlike plants and microorganisms. Most importantly, it was also reported for its biological activity properties and its potential as a capping agent (Ismail et al., 2019). Eucalyptus flower species is usually one of the main sources of nectar for the Australian honeybees (*Apis mellifera*) to produce honey. The source of nectar, the combination of proteins secreted by the bee for the honey-ripening process, and protein from plant pollen will affect the honey’s chemical composition. According to Beiranvand et al. (2021), the major component in pure Australian honey was carbohydrates, which could act as a capping and reducing agent. However, the chemical component such as carbohydrate in honey is considered a weak reducing agent so it needs another booster to enhance the reducing process of the nanoparticles, and for this, vitamin C (ascorbic acid) was chosen in this study since it is also a green material. Hence, in this work, we produced a honey-mediated copper reduced graphene oxide (H/Cu/RGO) nanocomposite using a sonochemical method, where Australian honey and ascorbic acid were served as capping and reducing agents, respectively, during the process. The sample was analyzed by using UV-visible, XRD, HRTEM, and FTIR, and it was then tested for antibacterial and cytotoxicity properties.

## Materials and methods

### Materials

The source of honey was from the Capilano Honey Limited (Australia). Standard graphene oxide (water dispersion, 4 mg/ml) was purchased from Graphene (U-Malaya). Copper II nitrate trihydrate (Cu(NO_3_)_2_.3H_2_O, AR grade), ascorbic acid (C_6_H_8_O_6_, AR grade), and sodium hydroxide (NaOH) were purchased from R&M Chemical, United Kingdom. All the chemicals were of analytical grade without further purification. Two Gram-positive bacteria, methicillin-resistant *Staphylococcus aureus* (MRSA, clinical isolate) and *Enterococcus faecalis* (*E. faecalis*, ATCC 33186), and two Gram-negative bacteria, *Escherichia coli* (*E. coli*, ATCC 11775) and *Pseudomonas aeruginosa* (*P. aeruginosa*, ATCC 10145), were used for antibacterial assessment. They were cultured and maintained in sterile Mueller–Hinton agar and broth media (Becton Dickinson, United States). The colorectal cancer cell line HCT116 (ATCC CCL-247) and human normal colon cell CCD112 (ATCC CRL-1541) were used for the cytotoxicity assay.

### Synthesis of reduced graphene oxide (RGO) and honey-mediated copper/reduced graphene oxide (H/Cu/RGO) nanocomposite

RGO was produced through the reduction of graphene oxide. For this, 2 ml of GO was added to 50 ml of deionized water. After vigorous stirring for around 30–40 min, 7.5 ml ascorbic acid (1 M) was slowly added to the solution using a dropper, while the sample was treated with ultrasonic irradiation for 10 min with a fixed setting parameter (amplitude 80%, pulse on 1s and pulse off 1 s). The mixture was then cooled down to room temperature (RT), centrifuged, washed with distilled water, and dried in the oven. The H/Cu/RGO nanocomposite was synthesized following the method by Zhang et al. (2016) with some modifications. In brief, Australian honey (15 w/v %) was dissolved in 50 ml of 0.025 M Cu(NO_3_)_2_.3H_2_O, and the pH was adjusted between pH 7 to 8. Afterward, 1 ml of the RGO (1.5 mg/ml) was mixed with the combination of honey and copper salt solution under continuous stirring at RT for 30–40 min. The mixture solution was then treated with ultrasonic irradiation for 10 min by adding 7.5 ml of 1 M ascorbic acid dropwise simultaneously. The compound was finally cooled down to RT, centrifuged, washed with distilled water, and dried in the oven.

### Characterizations of the H/Cu/RGO nanocomposite

The synthesis H/Cu/RGO nanocomposite was determined using ultraviolet-visible (UV-vis) spectroscopy (UV-2600, SHIMADZU) in the range of 220–800 nm. X-ray diffraction (XRD, Philips, X’pert, Cu Ka) was used to analyze the structure of the H/Cu/RGO nanocomposite in the range of 5°–80° (2θ). The size and the shape of the nanocomposite were evaluated by using high-resolution transmission electron microscopy (HRTEM, JEM-2100F). Fourier transform infrared (FTIR) spectra were obtained using an attenuated total reflectance (ATR) IRTracer-100 spectrophotometer (Shimadzu, Malaysia). The spectra were set within a range of 400–4,000 cm^−1^.

### Antibacterial activity

To determine the minimum inhibitory concentration (MIC) values, the broth micro-dilution method was used for the H/Cu/RGO nanocomposite against Gram-positive (MRSA and *E. faecalis*) and Gram-negative (*E. coli* and *P. aeruginosa*) bacteria using the Clinical and Laboratory Standards Institute (CLSI) protocols. For this, a single colony of fresh bacterial culture (12–18 h) was isolated from the Mueller–Hinton agar (MHA) plates and inoculated into the Mueller–Hinton broth (MHB). The culture was grown overnight (16–18 h) prior to the experiments. The next day, the bacterial concentration was standardized to an optical density (OD) of 600 nm (approximately 1 × 10^8^ CFU/ml) with MHB. Two-fold serial dilutions of the H/Cu/RGO nanocomposite were prepared in 96-well plates to get the final test concentrations of 0, 7.8, 15.6, 31.3, 62.5, 125, 250, 500, and 1,000 μg/ml per well. Thereafter, 10 μl of bacterial suspension equivalent to 10^6^ CFU/ml of exponentially growing bacterial cells were added to the wells followed by 18 h of incubation at 35 ± 2°C. The plate was then read for absorbance at 600 nm using a microplate reader (GloMax Discover Instrument, Promega). The percentage of cell viability was calculated using Equation (1), and the minimum inhibitory concentration which inhibits 50% bacterial growth (MIC_50_) value was then determined.

%Viability=OD of sample well (mean)/OD of control well (mean)×100. (1)

### Cytotoxic effect of the H/Cu/RGO nanocomposite

Cell proliferation assay (Promega) was used to determine the cytotoxic properties of the H/Cu/RGO nanocomposite. Briefly, 5 × 10^3^ human colorectal cancer cell line HCT116 and human normal colon cell CCD112 were seeded in a 96-well plate (100 μL/well) and incubated at 37°C overnight in a 5% CO_2_ humidified incubator. The next day, 2-fold serially diluted nanocomposites (500, 250, 125, 62.5, 31.3, 15.6, 7.8, and 0 μg/ml) were added into the wells (100 µl/well). After 72 h incubation at 37°C in a 5% CO_2_ humidified incubator, the wells were treated with 20 µl MTS (3-(4,5-dimethylthiazol-2-yl)-5-(3-carboxymethoxyphenyl)-2-(4-sulfophenyl)-2H-tetrazolium) reagent followed by an additional 3 h incubation at 37°C in the 5% CO_2_ incubator. Optical density (OD) was then measured at 490 nm using a multimode microplate reader (Tecan). The dose–response graph was plotted by calculating the percent of cell viability using Eq. 1, and half maximal inhibitory concentration (IC_50_) was then calculated.

## Results and discussion

### Synthesis of the H/Cu/RGO nanocomposite

**SCHEME 1:**
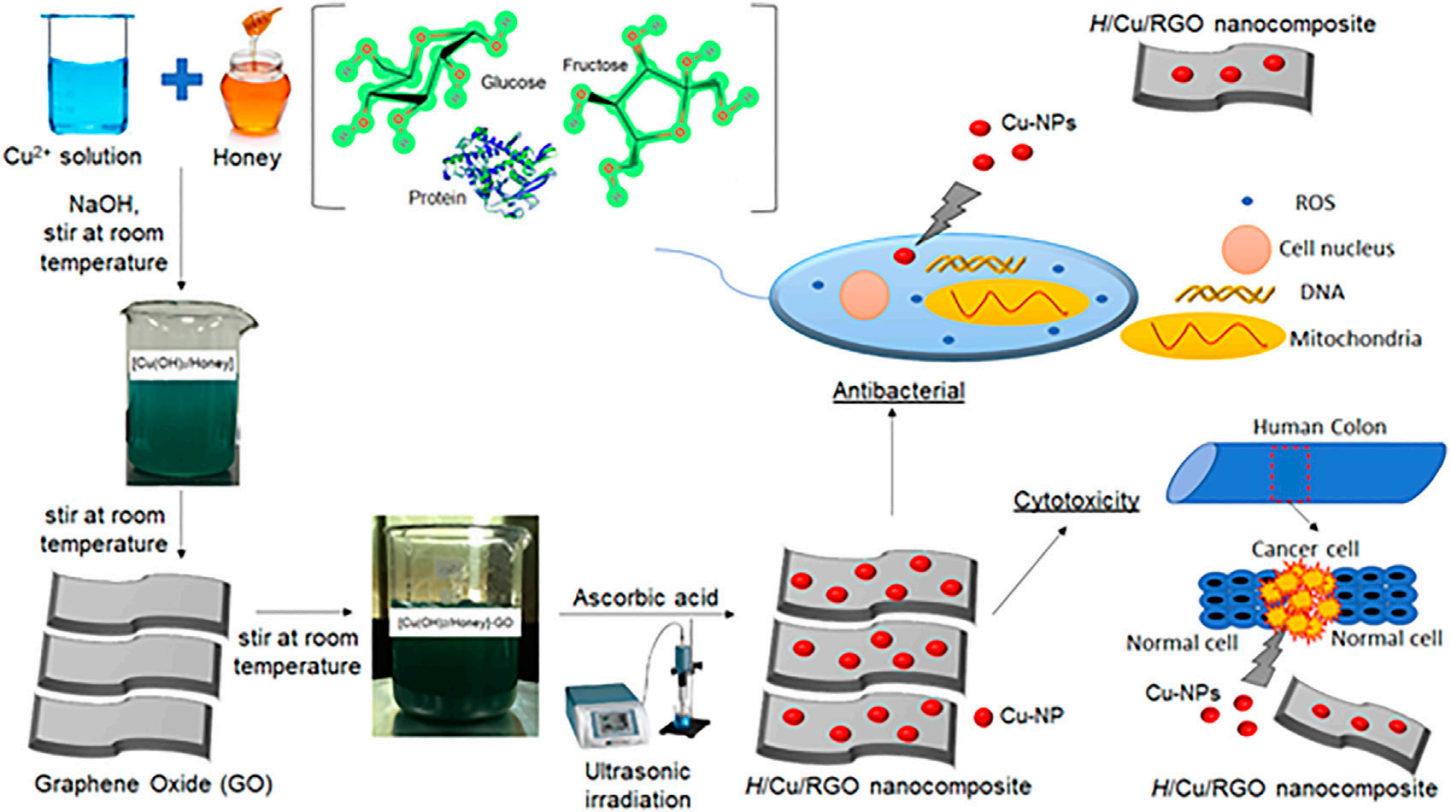
Schematic illustration of H/Cu/RGO nanocomposite synthesis and its applications.

As illustrated in Scheme 1, we used copper nitrate solution as a precursor for Cu-NPs synthesis. To accelerate the process, NaOH was added to form an intermediate which is copper hydroxide Cu(OH)_2_. The pH of the solution was controlled between pH 7 to 8 since it is the preferred environment to produce smaller sizes of pure Cu-NPs. According to Amjad et al., when the pH increased (between pH 6 and pH 10), the size of nanoparticles decreased (Rajesh et al., 2016; Amjad et al., 2021). Since the aim of this study is to produce pure metallic Cu-NPs, the pH needs to be in a basic medium. The reduction process of the compound was furthered with the addition of the ascorbic acid as a reducing agent and assistance of ultrasonic irradiation to enhance the reaction process. Honey acts as a capping agent to control the size and shape of the nanoparticles in the solution. The nanocomposite was then tested against the bacteria and cancer cell line to observe its biological activities.

Meanwhile, Eqs 2–5 described the possible chemical formation of H/Cu/RGO nanocomposite.

$$Cu^{2+}\left(\mathrm{aq}\right)+\mathrm{Honey}\left(1\right)\overset{\mathrm{Stirring}}{\underset{T=25\;{}^{\circ}C}{\to }}{\left[\mathrm{Cu}\left(\mathrm{Honey}\right)\right]}^{2+}\left(\mathrm{aq}\right),$$ (2)

$${\left[\mathrm{Cu}\left(\mathrm{Honey}\right)\right]}^{2+}\left(\mathrm{aq}\right)+2OH^{-}\left(\mathrm{aq}\right)\overset{\mathrm{Stirring}}{\underset{T=25\;{}^{\circ}C}{\to }}\left[\mathrm{Cu}{\left(\mathrm{OH}\right)}_{2}/\mathrm{Honey}\right]\left(s\right),$$ (3)

$$\left[\mathrm{Cu}{\left(\mathrm{OH}\right)}_{2}/\mathrm{Honey}\right]\left(\mathrm{aq}\right)+\mathrm{GO}\left(\mathrm{aq}\right)\overset{\mathrm{Stirring}}{\underset{T=25\;{}^{\circ}C}{\to }}\left[\mathrm{Cu}{\left(\mathrm{OH}\right)}_{2}/\mathrm{Honey}\right]-\mathrm{GO}\left(\mathrm{aq}\right),$$ (4)

$$\left[\mathrm{Cu}{\left(\mathrm{OH}\right)}_{2}/\mathrm{Honey}\right]-\mathrm{GO}\left(\mathrm{aq}\right)+2H_{2}O\left(1\right)\overset{C_{6}H_{8}O_{6}}{\underset{\mathrm{Ultrasonic}\;\mathrm{Irradiation},\;t=10}{\to }}\left[H/\mathrm{Cu}/\mathrm{RGO}\;\mathrm{nanocomposite}\right]\left(\mathrm{aq}\right)+C_{6}H_{6}O_{6}\left(\mathrm{aq}\right).$$ (5)

The formation of the reaction process of the Cu(OH)_2_/honey complex (Eq. 2) resulted in a blue-colored solution as previously reported study (Ismail et al., 2019). Cu(OH)_2_ was obtained by adding the sodium hydroxide (NaOH), and it acts as nuclei during the process. A mutarotation process occurs during this phase since OH^−^ in the solution could change the α-glucose into β-glucose by opening the chain structure and forming the aldehyde group (-CHO) (Upadhyay and Kumar, 2017; Alejandro et al., 2017). This aldehyde group with the presence of the energy from the ultrasonic irradiation process was then oxidized by the complex copper ions to form the gluconic acid. This initiates the nucleation and growth of the Cu-NPs in the solution. In addition, the Cu^2+^ ion growth could also occur at the nucleation site of the GO substrate as the reduction of Cu^2+^ takes place through galvanic displacement and redox reaction (Eq. 4). The GO and copper ions were further reduced to H/Cu/RGO nanocomposites with the presence of ascorbic acid and the assistance of the ultrasonic irradiation process. It could be concluded that GO acts as a substrate and could also help as a reducing agent for the copper ions (Zhang et al., 2016). The GO sheets might bind with the copper ion by electrostatic interaction of the copper ion and through the cation-pi (cation-) interaction of the benzene ring with the cation (Cu^2+^) (Alayande et al., 2020).

### Characterization of GO, RGO, and H/Cu/RGO nanocomposite

**FIGURE 1:**
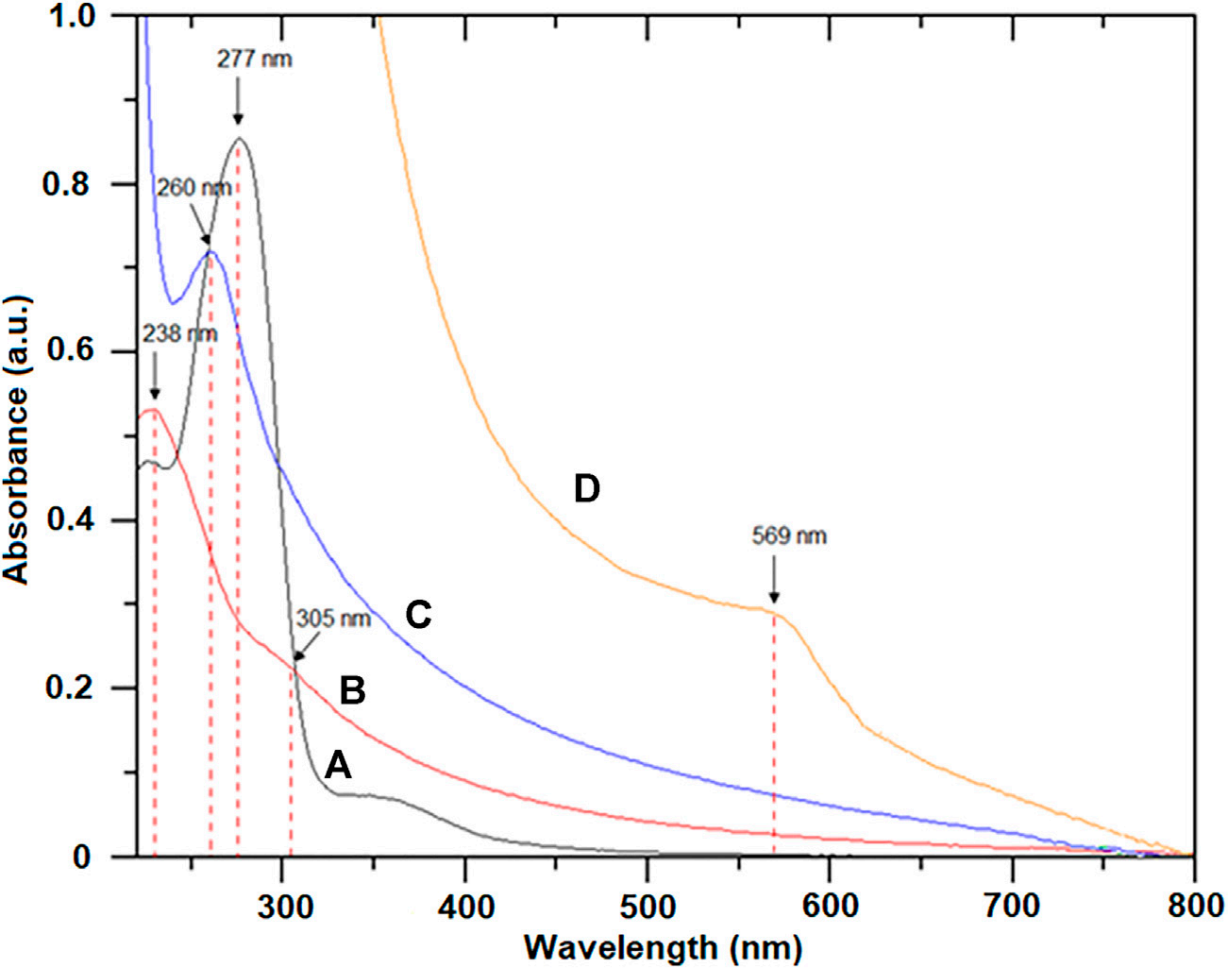
UV-visible spectra of **(A)** honey, **(B)** GO, **(C)** RGO, and **(D)** H/Cu/RGO nanocomposite.

Figure 1 shows the UV-vis spectra of the honey, GO, RGO, and H/Cu/RGO nanocomposite samples. The absorption peak of the honey appeared around 277 nm due to the origin and age of the honey itself (Posudin, 2016; Zhang et al., 2016). The π−π* transition of the C=C bond and n– π* transition of the C=O bond could be seen in Figure 1B for GO at 238 and 305 nm, respectively. The shifting peak at 260 nm to a higher wavelength and the peak around 305 nm disappeared for the RGO (Figure 1C) indicating the reduction of GO to RGO by the ascorbic acid. This phenomenon occurred because of the restoration of the aromatic system conjugation and the decrease of the carboxyl groups in the RGO layer (Navya Rani et al., 2019; Kang et al., 2020). The higher the conjugation degree, the lower the energy required in order to produce the electronic transition, and hence, the peak is shifted to a higher value associated with less energy involved (Rios et al., 2019). Figure 1D depicts the surface plasmon resonance of metallic phase copper nanoparticles (Cu-NPs) exhibited at 569 nm which proved the reduction of Cu^2+^ to Cu^0^ occurs during the synthesis process (Fahiminia et al., 2019).

**FIGURE 2:**
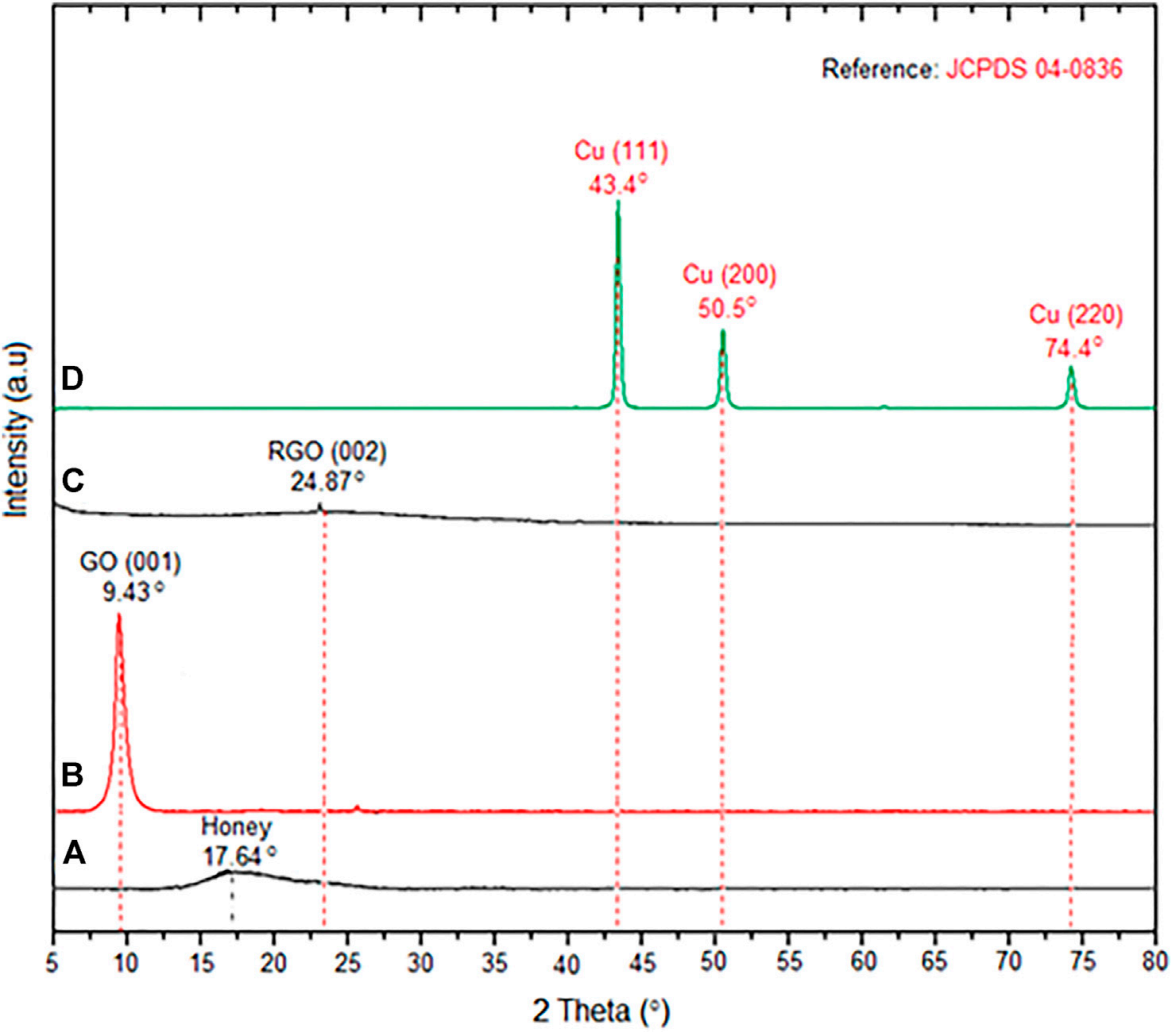
XRD patterns of **(A)** honey, **(B)** GO, **(C)** RGO, and **(D)** H/Cu/RGO nanocomposite.

XRD analysis was conducted for the honey, GO, RGO, and H/Cu/RGO nanocomposite. Figure 2A shows that the XRD diffraction pattern of honey at 2θ = 17.64° with a broad peak. The shifting of the diffraction peak in Figures 2B,C for GO and RGO from 9.43° to 24.87° indicated that the reduction of GO to RGO occurred under sonication treatment with the presence of the ascorbic acid. The interspacing distance between the layer of the GO and RGO was calculated by using Bragg’s law equation as in Eq. 6

nλ=2d⁡sin⁡θ, (6)

where *n* = 1, λ is the wavelength of the X-ray beam (0.154 nm), d is the distance between adjacent GO or RGO sheets, and θ is Bragg’s angle. The values of interspacing distance were 0.937 and 0.357 nm, respectively, for GO and RGO. The decrement of the value shows that the formation of RGO occurs according to the previous study (Rana et al., 2018). The changes in the interspacing distance reveal the exfoliation of the RGO layer happened after the reduction process and the decrease of the oxygenated functional groups on the surface (Kumar et al., 2019). For the H/Cu/RGO nanocomposite (Figure 2D), three diffraction peaks at 2θ = 43.4°, 50.5°, and 74.4° could be assigned to the (111), (200), and (220) crystal planes corresponding to the cubic structure of Cu which signified the formation of metallic copper on the RGO. These diffraction peaks of Cu were matched with the standard reference of the metallic Cu for the cubic structure which is JCPDS 04-0836. The peak for RGO could not be seen in the XRD pattern of the H/Cu/RGO nanocomposite, which could be related to the aggregation and restacking layer of the RGO with the insertion of the Cu-NPs in the nanocomposite (Guo et al., 2016; Kumar et al., 2019; Chen et al., 2020).

**FIGURE 3:**
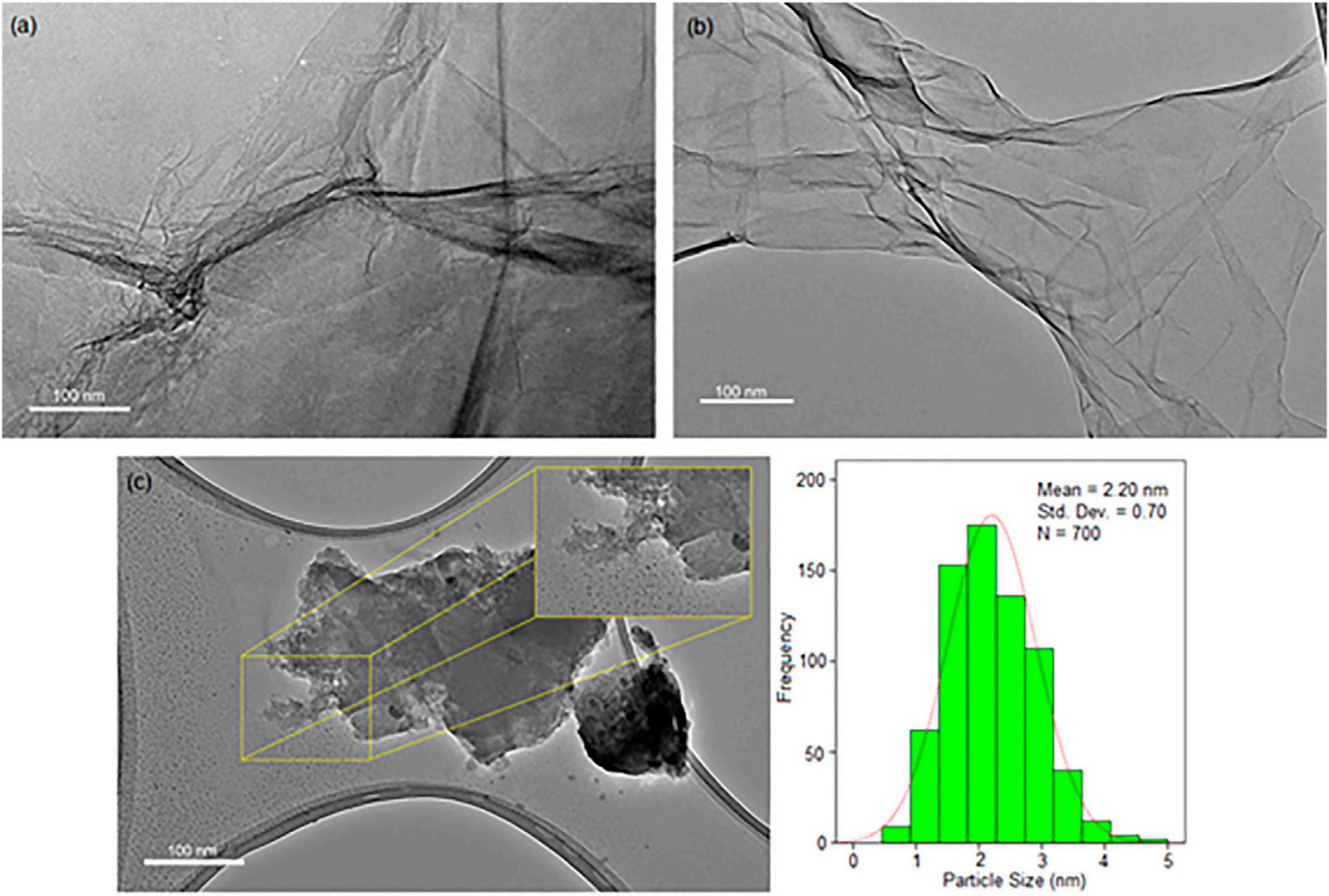
HRTEM images of **(A)** GO, **(B)** RGO, and **(C)** H/Cu/RGO nanocomposite.

In the HRTEM images (Figure 3), GO showed a fine-layer structure like a sheet, while RGO revealed a wrinkled structure. This phenomenon is related to the reduction of the GO to RGO, where the GO layer was exfoliated and tended to be decreased in size as it was treated with the ultrasonic. The thermal treatment through the ultrasound irradiation process can lead to the wrinkling of the RGO due to the reduction of the amount of oxygen-containing functional groups during sheet exfoliation (Rana et al., 2018; Khan et al., 2020). The Cu-NPs in the nanocomposite formed uniform spherical particles with a size of 2.20 ± 0.70 nm on the RGO layers. The exfoliation of the RGO layers into smaller scale with a fine particle of Cu-NPs that either are decorated between or on the surface of RGO layers happened. It concludes that Cu-NPs can bind to the graphene-based materials to form Cu/RGO nanocomposite (Zhu et al., 2017; Menazea and Ahmed, 2020).

**FIGURE 4:**
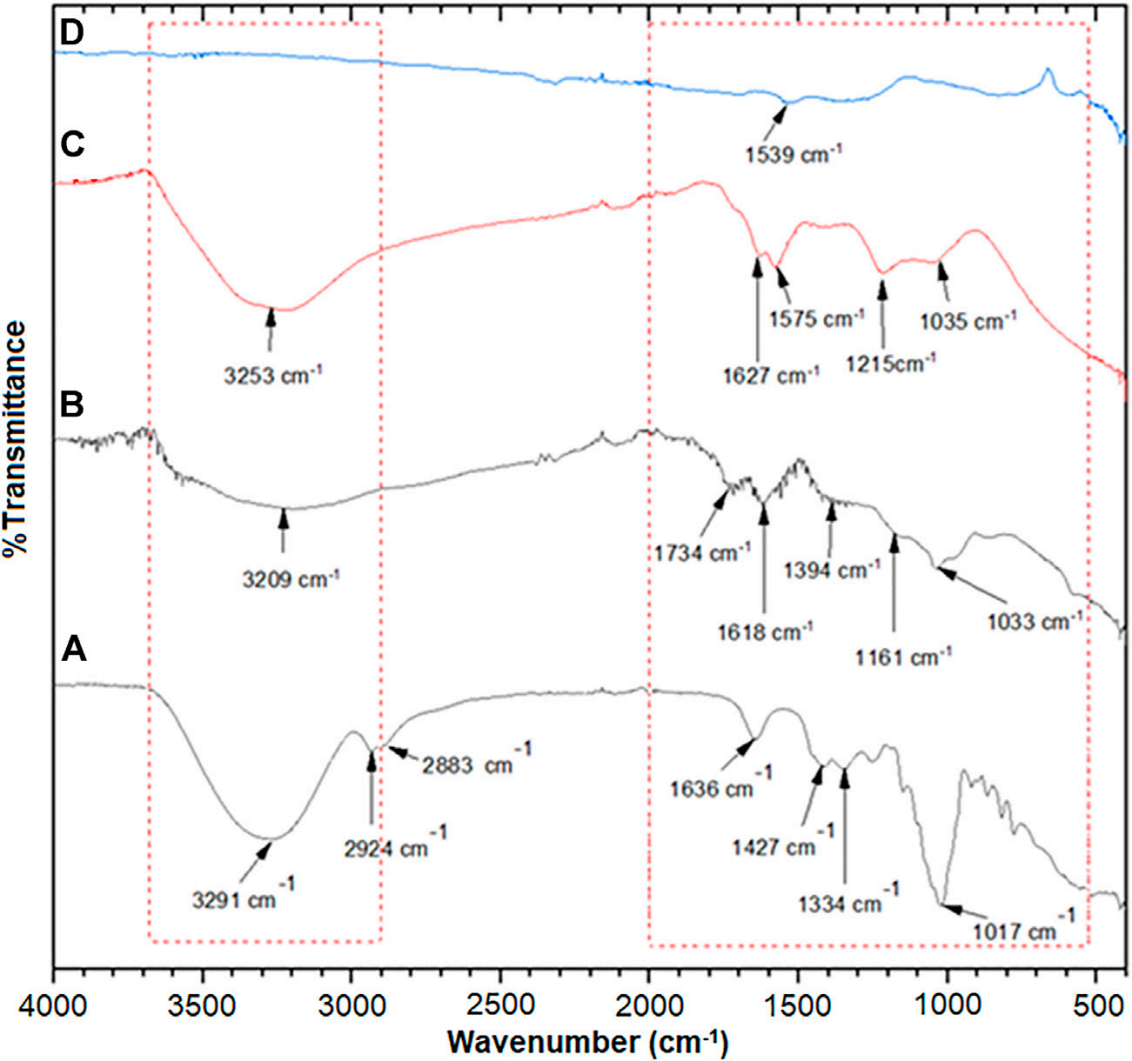
FTIR spectra of **(A)** honey, **(B)** GO, **(C)** RGO, and **(D)** H/Cu/RGO nanocomposite.

Through the FTIR spectra (Figure 4), the honey peak (Figure 4A) illustrated a strong and broad peak at 3,291 cm^−1^ related to a hydroxyl group (-OH) stretching vibration which also might overlap with the -NH stretching vibration of primary amine protein (Boldeiu et al., 2019). Two weak peaks appeared at 2,924 cm^−1^ and 2,883 cm^−1^, matched to the C-H stretching bands of the aldehyde group of glucose. Carbonyl group (-C=O) stretching vibration of protein could be seen at 1,636 cm^−1^, and the peak at 1,427 cm^−1^ and 1,334 cm^−1^ were related to C-H bending and C-O bending of glucose. While, at 1,017 cm^−1^, the peak correlated to C-O-C stretching, C-O stretching, and C-N stretching amine of glucose, fructose, and protein in honey. The GO spectrum (Figure 4B) demonstrated O-H stretching vibration with a broad peak between 3,200 cm^−1^ to 3,600 cm^−1^. The peaks at 1734, 1,618, 1,394, 1,161, and 1,033 cm^−1^ were correlated to the C=O stretching vibration of carbonyl groups presented in the GO sheet, C=C skeletal vibration, the sp^3^ C-H stretching vibration of saturated carbon, the epoxy C-O stretching vibration, and the alkoxyl C-O stretching vibration, respectively (Nguyen et al., 2019; Sengupta et al., 2019). However, in RGO, the disappearance of the carboxyl group at 1,734 cm^−1^ and sp^3^ C-H stretching vibration of saturated carbon at 1,394 cm^−1^ demonstrated the reduction of GO to RGO during the synthesis process. For the Cu/RGO nanocomposite (Figure 4D), the C=C vibration of the graphene skeleton peak could be observed at 1,539 cm^−1^, indicating that the GO was reduced to form the Cu/RGO nanocomposite (Navya Rani et al., 2019). In addition, the weak peak band area around 900 cm^−1^ in Figure 4D could be due to the shifting of the C-O/C-N stretching of protein and carbohydrate of honey biomolecules presented in the H/Cu/RGO nanocomposite (Ismail et al., 2019).

### Antibacterial activity of the H/Cu/RGO nanocomposite

**FIGURE 5:**
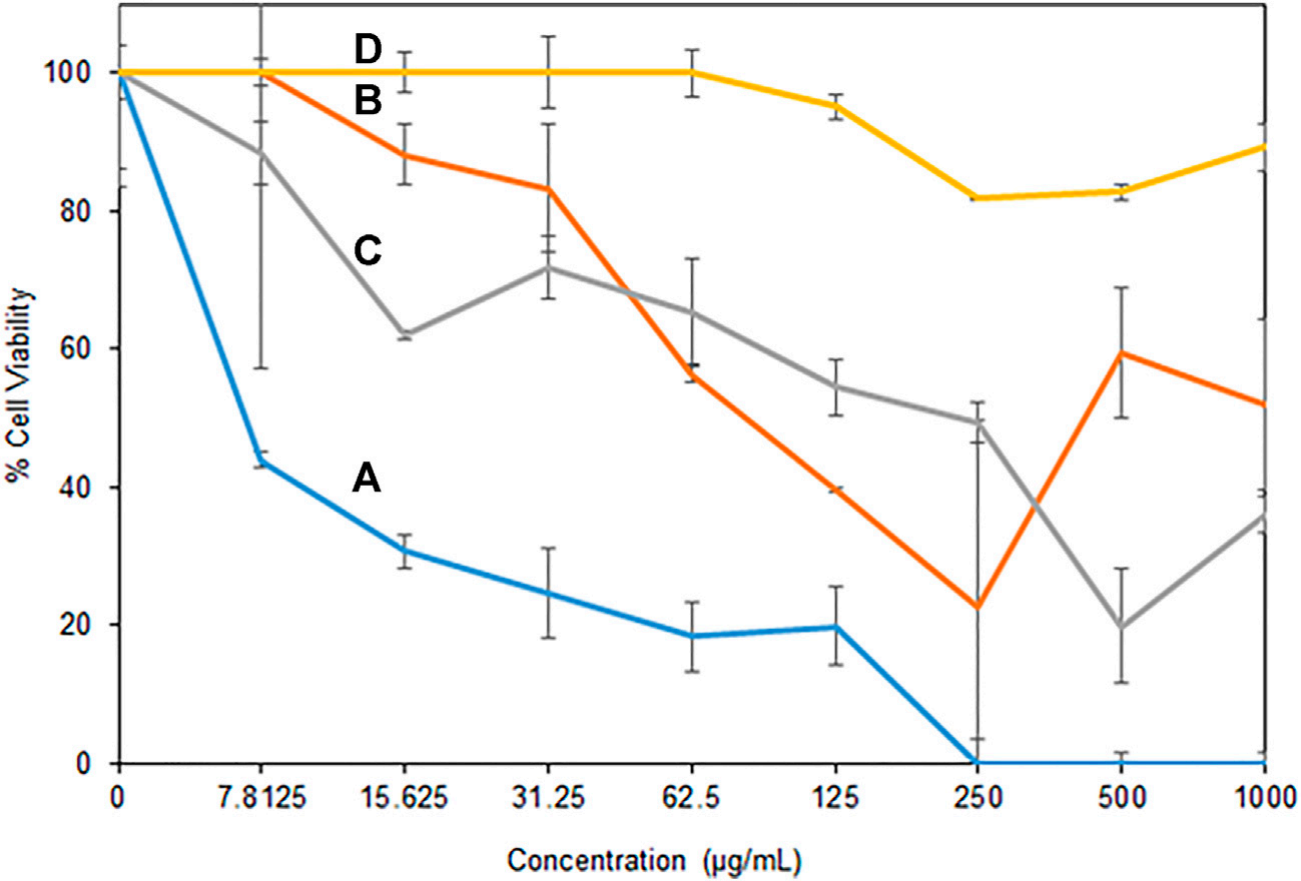
Antibacterial activity of *H*/Cu/RGO nanocomposite **(A)** *E. faecalis*, **(B)** MRSA, **(C)** *E. coli*, and **(D)** *P. aeruginosa*.

Antibacterial activity of the H/Cu/RGO nanocomposite toward bacterial strains was tested using the minimum inhibitory concentration (MIC) assay, where the selected Gram-positive (MRSA and *E. faecalis*) and Gram-negative (*P. aeruginosa* and *E. coli*) bacteria were found to be affected by the nanocomposite. Figure 5 illustrates that the inhibition activity of the nanocomposite was better toward Gram-positive bacteria compared to the Gram-negative strains. As shown in Table 1, the lowest MIC_50_ value was detected toward *E. faecalis*, where the nanocomposite could inhibit the growth of less than a quarter of the bacteria at a low concentration of 6.12 μg/ml, while for MRSA, the MIC_50_ was detected at the concentration of 67.96 μg/ml. For Gram-negative strains, the MIC_50_ was at 134.16 μg/ml and greater than 1,000 μg/ml for *E. coli* and *P. aeruginosa*, respectively.

**TABLE 1:** MIC_50_ values of H/Cu/RGO nanocomposite against four bacteria strains.

Sample	MIC_50_ of sample (µg/ml)			
	Bacterial strains			
H/Cu/RGO nanocomposite	*MRSA*	*E. faecalis*	*P. aeruginosa*	*E. coli*
	67.96	6.12	>1,000	134.16

The value of Cu-NPs as antibacterial agents has been studied for a long time (Lv et al., 2020). However, the H/Cu/RGO nanocomposite gave better antibacterial activity performance compared to the Cu-NPs only. In a prior study where honey-mediated Cu-NPs was tested on *E. faecalis* and *E. coli*, the values of MIC_50_ were 15.6 μg/ml and 250 μg/ml, respectively, which were higher than the value gained from the nanocomposite in current work. This is most possibly due to the toxic effects of Cu-NPs and the RGO that influenced the bacteria cells. It might be due to the Cu-NPs that affect bacteria by the generation of reactive oxygen species, lipid peroxidation, protein oxidation, and DNA degradation through liberating nascent Cu ions from the Cu-NP surface (Chatterjee et al., 2014). The cell membrane stress due to the graphene sheet layer structure itself is also possibly among the factors for the bactericidal activity to change (Prasad et al., 2017).

A possible explanation for the variation in antibacterial activity against different bacterial strains can be related to the difference in the bacteria cell envelope (Sriramulu et al., 2020). In contrast to Gram-positive strains which consist of the layers of peptidoglycan, Gram-negative bacteria, besides the inter thin peptidoglycan cell wall, are surrounded by an outer membrane containing lipopolysaccharide (LPS), which can act as an additional protection shield for the cell. It could be one of the possible reasons why *P. aeruginosa* could tolerate the H/Cu/RGO nanocomposite, even at high concentrations.

### Cytotoxic effect of the H/Cu/RGO nanocomposite

**FIGURE 6:**
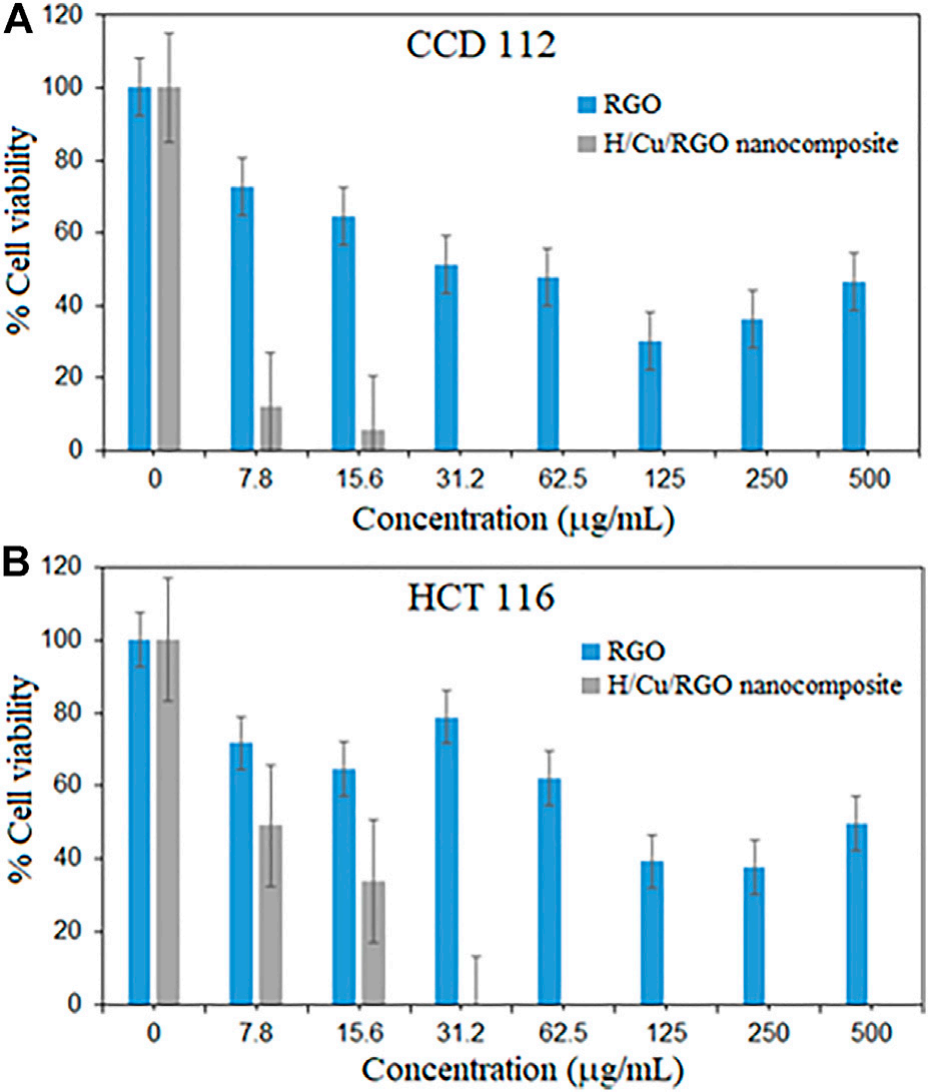
Cytotoxicity activity of *H*/Cu/RGO nanocomposite. **(A)** Normal colorectal cell line (CCD1 112). **(B)** Colorectal cancer cell line (HCT 116).

Cytotoxic activity of the nanocomposite was tested in both normal and cancer colorectal cell lines (Figure 6), where the H/Cu/RGO nanocomposite showed a higher cytotoxic effect compared to RGO, even at low concentrations. This could be due to the combination of Cu-NPs and RGO that enhanced the properties of the cytotoxic activity. This enhancement might also be attributed to the size of the Cu-NPs attached to the RGO, which is smaller in size with a spherical shape that makes them easier to interact with the cells and kill them. Similar to the antibacterial study, in comparison with our previous work, the H/Cu/RGO nanocomposite showed higher anticancer action (IC_50_–7.7 μg/mL as shown in Table 2) than honey-mediated Cu-NPs without RGO (IC_50_–46.11 μg/ml) in HCT116 cells (Ismail et al., 2019). A previous study reported that Cu-NPs killed SW480 human colon cancer cells at an IC_50_ value of 68 μg/ml by inducing reactive oxygen species (ROS)-mediated apoptosis (Ghasemi et al., 2022). As this is the first study reporting potential anticancer action of the H/Cu/RGO nanocomposite, their exact mechanisms of cancer cell killing are not known and warrant further investigations.

**TABLE 2:** IC_50_ of the RGO and nanocomposite toward normal and cancer cell lines of the colorectal cell.

Samples	IC_50_ of sample (µg/ml)	
	CCD 112 (normal cell)	HCT 116 (cancer cell)
RGO	33.70	141.50
H/Cu/RGO nanocomposite	2.14	7.7

In both graphs (Figures 6A,B), at a concentration of 31.2 μg/ml, the nanocomposite killed both cells at 100%, which indicates that this compound is not selective toward cancer cells. This could be seen in Table 2, where both RGO and H/Cu/RGO nanocomposite are non-selective toward cancer cells. Thus, further modifications are needed to enhance the selectivity of the nanocomposite toward cancer cells. For example, the nanocomposite can be conjugated to target-specific aptamers, peptides, antibodies, or other ligands to allow the nanocomposite to specifically bind to the surface molecules of cancer cells and enhance the drug localization, retention effect, and cellular uptake (Sutradhar and Amin, 2014; Martinelli et al., 2019).

## Conclusion

In conclusion, the green synthesized Cu/RGO nanocomposite using honey and ascorbic acid as capping and reducing agents, respectively, resulted in small-sized and spherical-shaped Cu-NPs attached to the RGO sheet. Our data proved that the agglomeration of copper could be prevented by combining the Cu-NPs with graphene-based materials in the presence of eco-friendly capping and reducing agents. The nanocomposite revealed good antibacterial and cytotoxicity activities, making them suitable for biomedical applications. However, the nanocomposite needs to be further studied to improve its specificity toward cancerous cells.

## Data Availability

The original contributions presented in the study are included in the article/Supplementary Material; further inquiries can be directed to the corresponding authors.
